# Comparison between different advanced cannulation techniques for difficult biliary cannulation: a systematic review with a meta-analysis

**DOI:** 10.3389/fmed.2024.1344644

**Published:** 2024-04-23

**Authors:** Liangjing Wang, Limin Wang, Nan Huang, Tian Li, Xiaohua Shi, Qilin Zhu

**Affiliations:** ^1^Department of Gastroenterology, Suzhou Hospital, Affiliated Hospital of Medical School, Nanjing University, Suzhou, China; ^2^Department of Gastroenterology, Qingdao University of Internal Medicine, Qingdao, Shandong, China; ^3^Department of Nursing, HZ Domestic Professional College, Heze, China; ^4^School of Basic Medicine, Fourth Military Medical University, Xi’an, China

**Keywords:** double guide wire technique, endoscopic retrograde cholangiopancreatography, transpancreatic sphincterotomy, Success rate, meta-analysis

## Abstract

**Objective:**

This study aimed to systematically evaluate the efficacy and safety of the double-guidewire technique along with other methods (persistent standard cannulation techniques, transpancreatic sphincterotomy, and pancreatic stent-assisted technique) for difficult biliary cannulation.

**Methods:**

Two researchers searched for literature on the efficacy and safety of the double-guidewire technique and other techniques in difficult biliary cannulation in databases, including PubMed, Embase, Cochrane, China National Knowledge Infrastructure, and Wanfang Data, based on the inclusion and exclusion criteria. The success rate of cannulation, duration of cannulation, post-ERCP pancreatitis, and overall postoperative complications were also analyzed using RevMan 5.4 software.

**Results:**

In total, 20 randomized controlled trial (RCT) studies involving 2008 participants were identified. The success rate of cannulation in the double-guidewire technique was much higher than that in persistent standard cannulation techniques [RR = 1.37, 95%CI (1.05, 1.79), *p* = 0.02]. However, it was lower than the success rate observed with transpancreatic sphincterotomy [RR = 0.89, 95%CI (0.81, 0.97), *p* = 0.01]. There was no significance in post-ERCP pancreatitis [RR = 1.09, 95% CI (0.85, 1.40), *p* = 0.49], overall postoperative complications [RR = 0.90, 95% CI (0.56, 1.45), *p* = 0.66], and duration of cannulation [SMD = -0.14, 95%C I (−1.43, 1.15), *p* = 0.83] between the double-guidewire technique and other techniques.

**Conclusion:**

This study demonstrated that the success rate of cannulation ranged from transpancreatic sphincterotomy to the double-guidewire technique and then to persistent standard cannulation techniques.

## 1 Introduction

Endoscopic retrograde cholangiopancreatography (ERCP) is an endoscopic technique used in the diagnosis and treatment of biliary and pancreatic diseases, especially the biliary disease. Selective biliary cannulation is the basis of the diagnosis and treatment of biliary diseases but still has a failure rate of 15%, even in advanced care centers (1). The double-guidewire technique (DGW) involves leaving one guidewire in the pancreatic duct (PD) while attempting cannulation of the bile duct (BD) with a second guidewire. There is a hypothesis that suggests that the first guidewire might reduce the angulation of the distal bile duct, thereby facilitating its cannulation. When selective bile duct cannulation fails several times, the guidewire might enter the pancreatic duct, which will increase the possibility of post-endoscopic retrograde cholangiopancreatography pancreatitis (PEP) (2). Most PEPs are mild, while severe acute pancreatitis might result in pancreatic necrosis, multiple organ failure, and death (3).

Many researchers have been focusing on the study of endoscopic surgery to prevent PEP. Compared to auxiliary cannulation, which requires the injection of contrast medium, DGW has a higher success rate of cannulation and a lower incidence rate of PEP (4). DGW applies to difficult biliary cannulation in ERCP. However, there are debates regarding the study of DGW and other techniques. This study aimed to compare DGW with other technologies, such as persistent standard cannulation techniques, transpancreatic sphincterotomy, and pancreatic stent-assisted technique, in the post-ERCP pancreatitis of difficult biliary cannulation. The success rate of cannulation, overall postoperative complications, and duration of cannulation will provide additional evidence for clinical research.

## 2 Materials and methods

### 2.1 Study design and search strategy

This study was registered in the PROSPERO database (CRD42023396158). The methodology is supervised by author Tian Li, a member of the Cochrane Collaboration. This study was conducted following the PRISMA checklist guidelines. We searched English databases, such as PubMed, Embase, and Cochrane, as well as Chinese databases, including China National Knowledge Infrastructure and Wanfang Data, from the initiation of the database to December 2023. The subject words were combined with free words, and sometimes, the reference was searched in order to increase the retrieval ratio. The Chinese keywords were “Pancreatic duct preincision technique,” “Transpancreatic sphincterotomy,” “Pancreatic guide wire inserted technique,” “Double guidewire technique,” “Preincision technique,” “Difficult biliary cannulation,” “persistent standard cannulation techniques,” “pancreatic stent-assisted technique,” “ERCP,” and “Acute pancreatitis.” English retrieval, taken PubMed as an example, the keywords were ((((((Double guidewire) AND (persistent standard cannulation techniques)) OR (Transpancreatic sphincterotomy)) OR (pancreatic stent-assisted technique)) OR (Transpancreatic precut)) OR (Transpancreatic septotomy)) AND (Difficult biliary cannulation).

### 2.2 Inclusion and exclusion criteria

The inclusion criteria include the following: (1) A randomized controlled trial (RCT) related to the double-guidewire technique and other techniques (persistent standard cannulation techniques, transpancreatic sphincterotomy, and pancreatic stent-assisted technique) in the post-endoscopic retrograde cholangiopancreatography (ERCP) pancreatitis of difficult biliary cannulation. The outcome indicators are related to the PEP, the success rate of cannulation, duration of cannulation, and overall postoperative complications.

The exclusion criteria include the following: (1) articles without peer-reviewe or those that are yet to be published; (2) repeated publications or incomplete data; and (3) the object of study was inconsistent with the intervention measures. The detailed PICOs are reported in Supplementary Table S1.

### 2.3 Data extraction and quality assessment

The literature related to the efficacy and safety of the double-guidewire technique and other techniques (persistent standard cannulation techniques, transpancreatic sphincterotomy, and pancreatic stent-assisted technique) in the post-ERCP pancreatitis of difficult biliary cannulation was searched by two researchers among various databases based on the inclusion and exclusion criteria. Literature extraction included the name of the first author, the country of the subjects, basic information about the subjects, the incidence of PEP, the success rate of cannulation, duration of cannulation, and the overall postoperative complications. If there were any discrepancies, we consulted a third senior investigator (K.W.). According to the Cochrane Systematic Review Manual, the quality of included literature studies were assessed as “low risk of bias,” “high risk of bias,” or “uncertain risk of bias.” The literature was evaluated on seven aspects: random selection, based on whether the assignment scheme was concealed, an implementation plan for blinding subjects and researchers, and an implementation plan for blinding result evaluators, data integrity, selective reporting, and other biases. Studies with more than 10 literature studies were used to analyze publication bias through a funnel plot.

### 2.4 Statistical analysis

All statistical analyses were performed using software RevMan 5.4. The chi-square test was used to analyze the heterogeneity among the included studies (*α* = 0.1). There was a significant heterogeneity among different studies when *p* ≤ 0.01 and *I^2^* > 50%. Relative risk (RR) was used for enumeration data, and standardized mean difference (SMD) was used for measurement data. *p*-values of <0.05 were considered significant.

## 3 Results

### 3.1 Literature search

**Figure 1:**
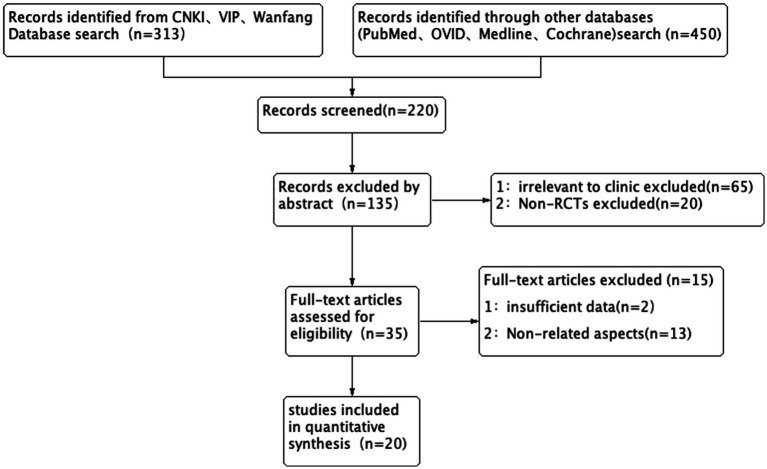
Flow diagram showing the study selection process for meta-analysis.

A total of 763 literature studies were searched by two researchers according to the inclusion and exclusion criteria. After removing duplicates, there were 220 studies for the review of titles and abstracts. During this process, 85 literature studies (65 studies with inconsistent intervention and control measures and 20 non-RCTs) were excluded. After reading the full text, 15 studies (13 studies with inconsistent outcome indicators and 2 studies with incomplete data) were excluded. Finally, 20 studies were included in the study (5–24), of which 11 were English literature studies (7–15, 23) and 9 were Chinese literature studies (5, 6, 16–22). There were 2,008 participants, of which 1,000 were in the DGW group and 1,008 were in the control group. In detail, 411 participants in the persistent standard cannulation techniques group, 517 in the transpancreatic sphincterotomy group, and 80 in the pancreatic stent-assisted technique group. The flowchart is shown in Figure 1, and the basic characteristics of the included literature are shown in Table 1.

**Table 1:** Characteristics of article.

Author	Country	Control group	Gender (Male/female)	Mean age (E/C)	Sample size (E/C)	Successful cannulation (E/C)	Cannulation time (E/C, min)	Complication (E/C)	PEP (E/C)
Wang et al. (5)	China	SCT	41/29	51.5^※^	34/36	32/11	NA	3/10	1/4
Zheng et al. (6)	China	SCT	82/9	41/43^※^	44/47	36/33	11.7 ± 3.2/16.8 ± 2.8	11/10	0/2
Herreros de Tejada et al. (7)	Spain	SCT	76/17	69.5/65.8^※^	97/91	46/51	NA	14/15	13/7
Maeda et al. (8)	Japan	SCT	23/30	64/64^※^	27/26	25/15	NA	0/0	0/0
Sasahira et al. (9)	Japan	SCT	141/133	NA	137/137	103/96	3.2 ± 2.8/3.4 ± 2.5	NA	27/23
Laquière et al. (10)	France	SCT	72/70	66.9/67.3^※^	68/74	57/37	NA	6/5	1/4
Kylänpää et al. (11)	Germany	TPS	98/105	68/66^※^	99/104	69/88	NA	21/17	16/14
Cha et al. (12)	Korea	TPS	NA	NA	39/42	31/39	NA	5/5	5/5
Mem et al. (13)	Egypt	TPS	18/22	56.16 ± 13.2/58.48 ± 17.8	19/21	18/20	20.1 ± 8.7/21.5 ± 7.8	NA	9/2
Sugiyama et al. (14)	Japan	TPS	37/31	67.3/69.8^※^	34/34	20/32	7.7 ± 0.7/9.5 ± 1.7	9/9	1/1
Yoo et al. (15)	Korea	TPS	41/30	63.7/67.0^※^	34/37	31/34	14.1 ± 13.2/15.4 ± 17.9	26/14	13/4
Li et al. (16)	China	TPS	35/33	62.44 ± 7.09/60.83 ± 7.11	34/34	23/32	NA	7/18	3/9
Yuan et al. (17)	China	TPS	37/32	49.2 ± 7.6/46.8 ± 8.3	35/34	18/24	7.83 ± 1.08/7.91 ± 1.20	NA	1/3
Tang et al. (18)	China	TPS	53/53	63.6 ± 5.7/64.9 ± 6.2	58/48	49/45	6.47 ± 1.84/5.29 ± 2.01	7/10	6/3
Lu et al. (19)	China	TPS	38/47	62.3 ± 3.4/61.7 ± 2.9	42/43	36/38	NA	NA	2/7
Sun et al. (20)	China	TPS	33/27	59.13 ± 6.54/58.53 ± 6.61	30/30	22/29	8.95 ± 2.52/4.83 ± 1.24	4/3	2/1
Wang et al. (21)	China	TPS	57/43	54 ± 7/54 ± 7	50/50	46/37	7.8 ± 1.2/7.5 ± 1.3	NA	1/6
Chen et al. (22)	China	TPS	43/39	62.5 ± 6.3/63.4 ± 5.3	42/40	34/36	NA	0/2	1/4
Coté et al. (23)	United States	PST	NA	57.4 ± 16.9/58.1 ± 17.2	42/45	16/26	17.0 ± 10.8/13.6 ± 15.9	1/3	1/3
Ito et al. (24)	Japan	PST	39/31	68/70^※^	35/35	33/28	NA	NA	8/1

E, experimental group; C, control group; SCT, standard cannulation techniques; PST, pancreatic stent-assisted technique; TPS, transpancreatic sphincterotomy technique; PEP, post-ERCP pancreatitis; NA, not available; ※: mean age.

### 3.2 Quality assessment of included studies

**Figure 2:**
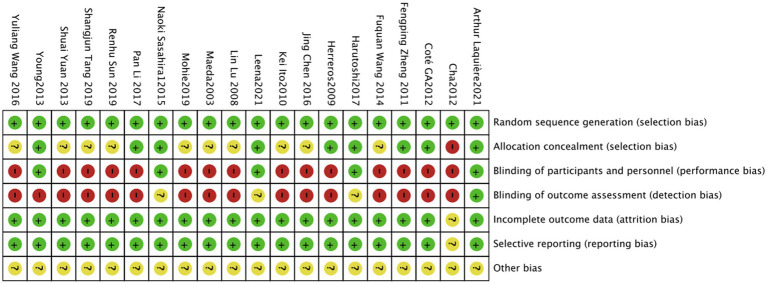
Risk-of-bias assessment.

**Figure 3:**
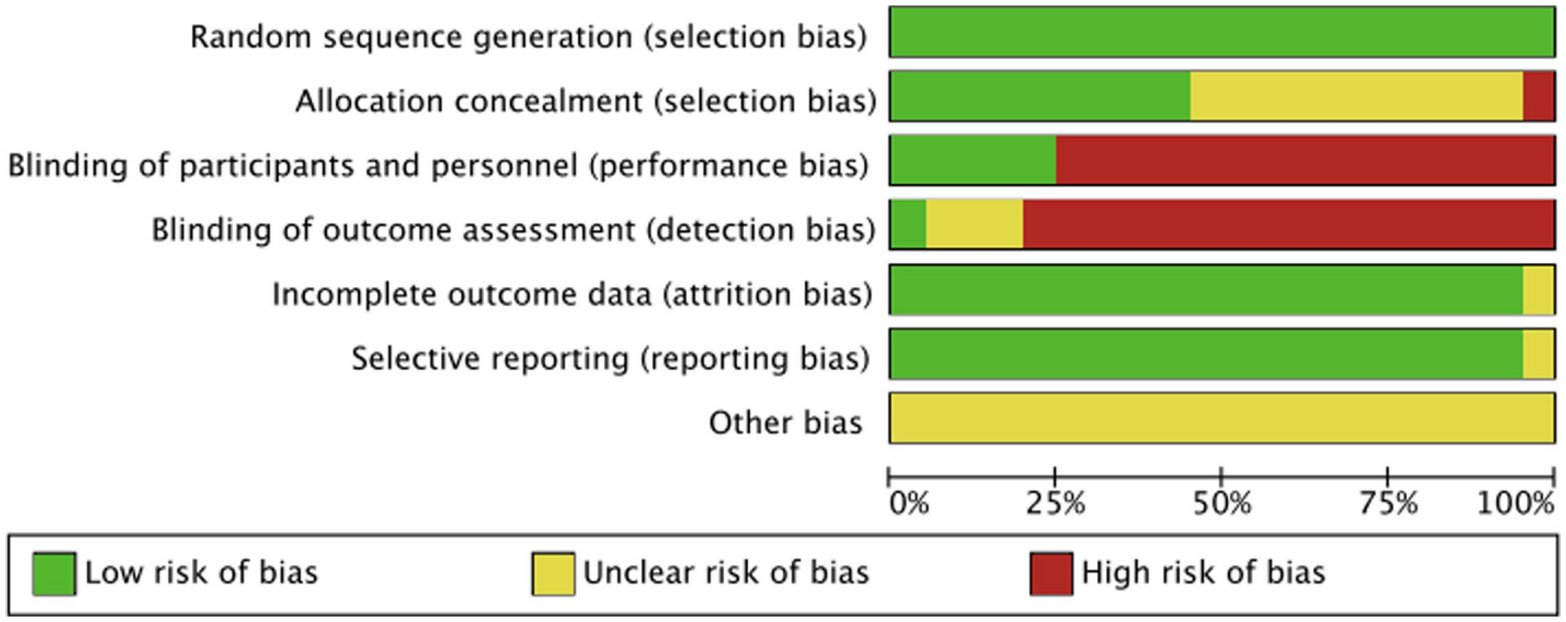
Proportion of risk bias in the included study literature.

Among the included studies, only nine (6, 7, 9–11, 14–16, 23) explained the random assignment scheme. One foreign literature could only obtain the abstract, with no mention of the concealment of the random assignment scheme and the blinding (12). The risk-of-bias assessment is shown in Figure 2, and the proportion of risk bias is shown in Figure 3.

### 3.3 The incidence rate of PEP

**Figure 4:**
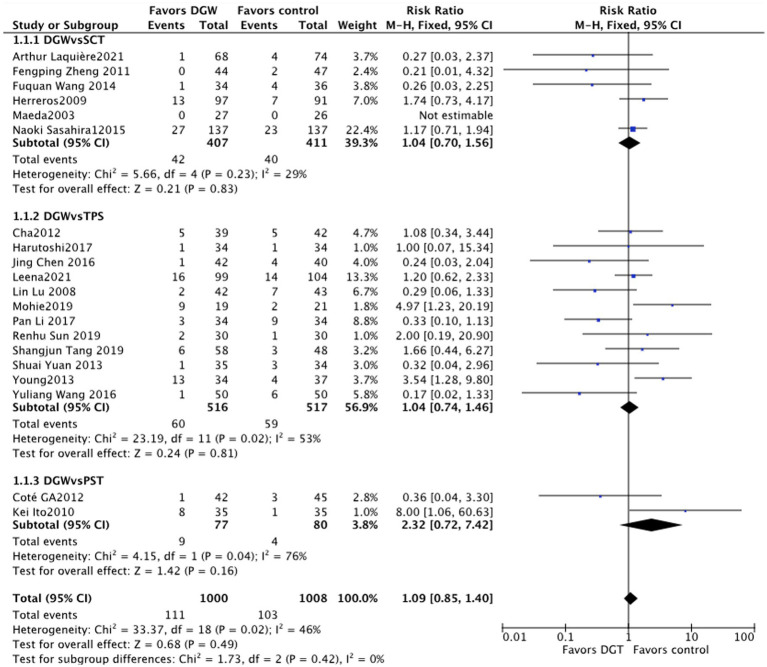
Post-endoscopic retrograde cholangiopancreatography (post-ERCP) pancreatitis: a meta-analysis of trials comparing the double-guidewire technique (DGW) vs. other endoscopic techniques, in patients with difficult cannulation.

In total, 20 studies (5–24) reported the incidence rate of PEP between DGW and other techniques, with no significant heterogeneity (*I*^2^ = 46%). The results of the meta-analysis demonstrated that there was no significant difference in the incidence rate of PEP between DGW and other techniques [RR = 1.09, 95% CI (0.85, 1.40), *p* = 0.49]. Additionally, there was no significant difference among the groups of the persistent standard cannulation techniques [RR = 1.04, 95% CI (0.70, 1.56), *p* = 0.83], transpancreatic sphincterotomy [RR = 1.04, 95% CI (0.74, 1.46), *p* = 0.81], and the pancreatic stent-assisted technique [RR = 2.32, 95% CI (0.42, 7.42), *p* = 0.16] (Figure 4).

### 3.4 Success rate of cannulation

**Figure 5:**
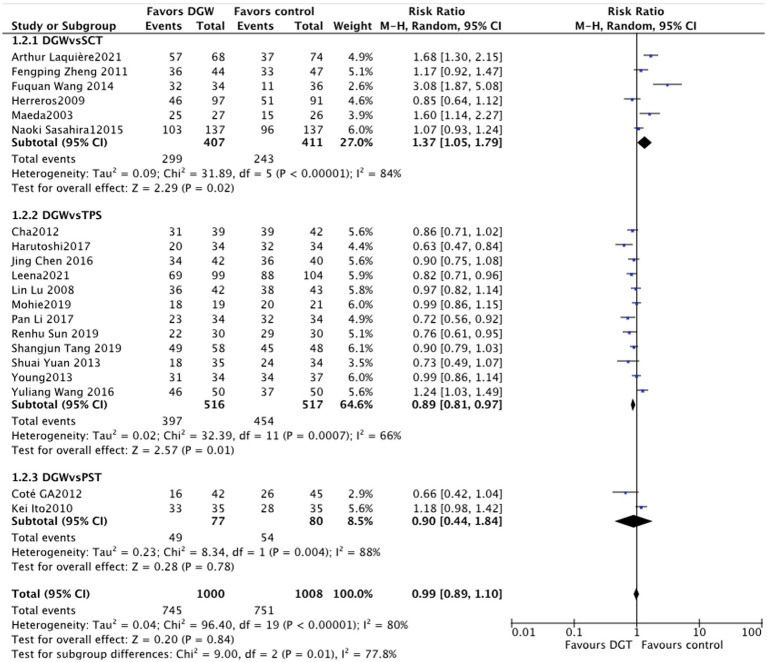
Overall success rate of cannulation: a meta-analysis of trials comparing the double-guidewire technique (DGW) vs. other endoscopic techniques, in patients with difficult cannulation.

In total, 20 studies (5–24) compared the success rate of ERCP for difficult biliary cannulation between DGW and other techniques, with significant heterogeneity among studies (*I*^2^ = 80%). The results showed that there was no significant difference in the success rate of cannulation between DGW and other techniques [RR = 0.99, 95%CI (0.89, 1.10, *p* = 0.84]. The success rate of cannulation in the transpancreatic sphincterotomy group was significantly higher than that in the DGW group [RR = 0.89, 95% CI (0.81, 0.97), *p* = 0.01]. Studies have indicated that placing a pancreatic duct stent before pancreatic tumor resection can prevent pancreatic duct injury and extend its surgical indications (25). Due to its specific anatomical structure, the placement of a pancreatic duct stent may potentially increase the success rate of bile duct cannulation. However, there was no significant difference between the DGW group and the pancreatic stent-assisted technique group [RR = 0.90, 95%CI (0.44, 1.84), *p* = 0.78] (Figure 5).

### 3.5 The incidence rate of overall postoperative complications

**Figure 6:**
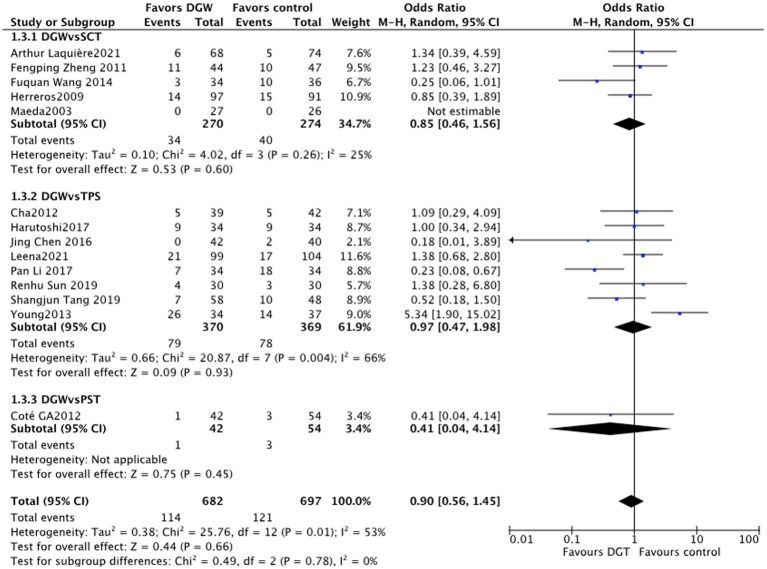
Overall complications: a meta-analysis of trials comparing the double-guidewire technique (DGW) vs. other endoscopic techniques, in patients with difficult cannulation.

In total, 14 studies (5–8, 10–12, 14–16, 18, 20, 22, 23) reported the incidence rate of overall postoperative complications of the ERCP difficult biliary cannulation between DGW and other techniques, with significant difference of heterogeneity (*I^2^* = 53%). There was no significant difference in the incidence rate of postoperative complications between DGW and other techniques [RR = 0.90, 95%CI (0.56, 1.45), *p* = 0.66]. In addition, the incidence rate of postoperative complications also had no significant difference among the persistent standard cannulation techniques group [RR = 0.85, 95%CI (0.46, 1.56), *p* = 0.60], the transpancreatic sphincterotomy group [RR = 0.97, 95%CI (0.47, 1.98), *p* = 0.93], and the pancreatic stent-assisted technique group [RR = 0.41, 95%CI (0.04, 4.14), *p* = 0.45] (Figure 6).

### 3.6 Duration of cannulation

**Figure 7:**
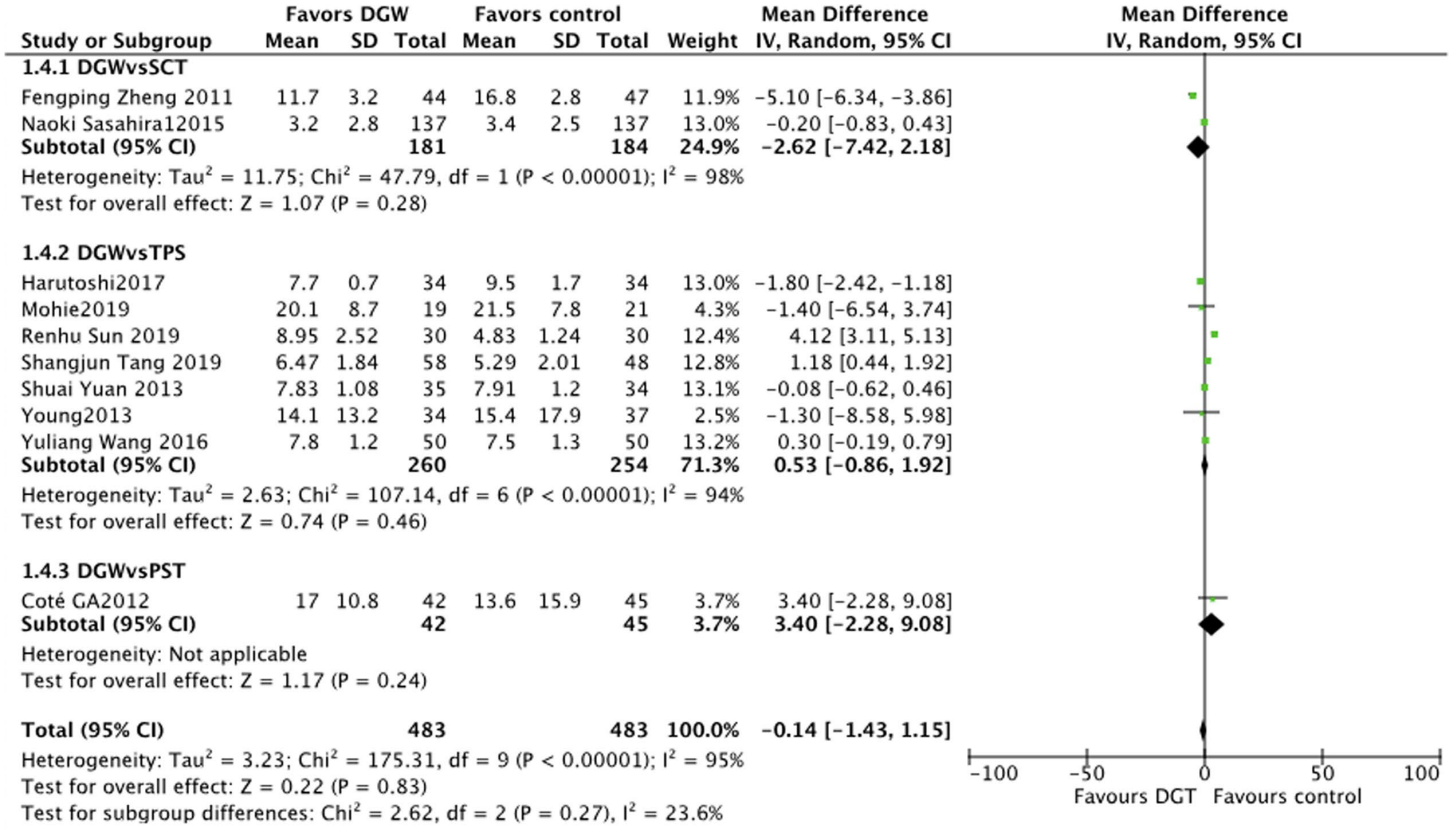
Duration of cannulation: a meta-analysis of trials comparing the double-guidewire technique (DGW) and other endoscopic techniques, in patients with difficult cannulation.

In total, 10 studies (6, 9, 13–15, 17, 18, 20, 21, 23) reported the duration of cannulation for ERCP difficult biliary cannulation between DGW and other techniques, with significant heterogeneity (*I^2^* = 95%). The results showed that there was no significant difference in the duration of cannulation between the DGW group and other technique groups [SMD = -0.14, 95% CI (−1.43, 1.15) *p* = 0.83]. Additionally, the duration of cannulation also showed no significant difference among the persistent standard cannulation techniques group [SMD = -2.62, 95% CI(−7.42, 2.18), *p* = 0.28], the transpancreatic sphincterotomy group [SMD = 0.53, 95% CI (−0.86, 1.92), *p* = 0.46], and the pancreatic stent-assisted technique group [SMD = 3.40, 95%CI (−2.28, 9.08), *p* = 0.24] (Figure 7).

### 3.7 Sensitivity analysis and publication bias

**Figure 8:**
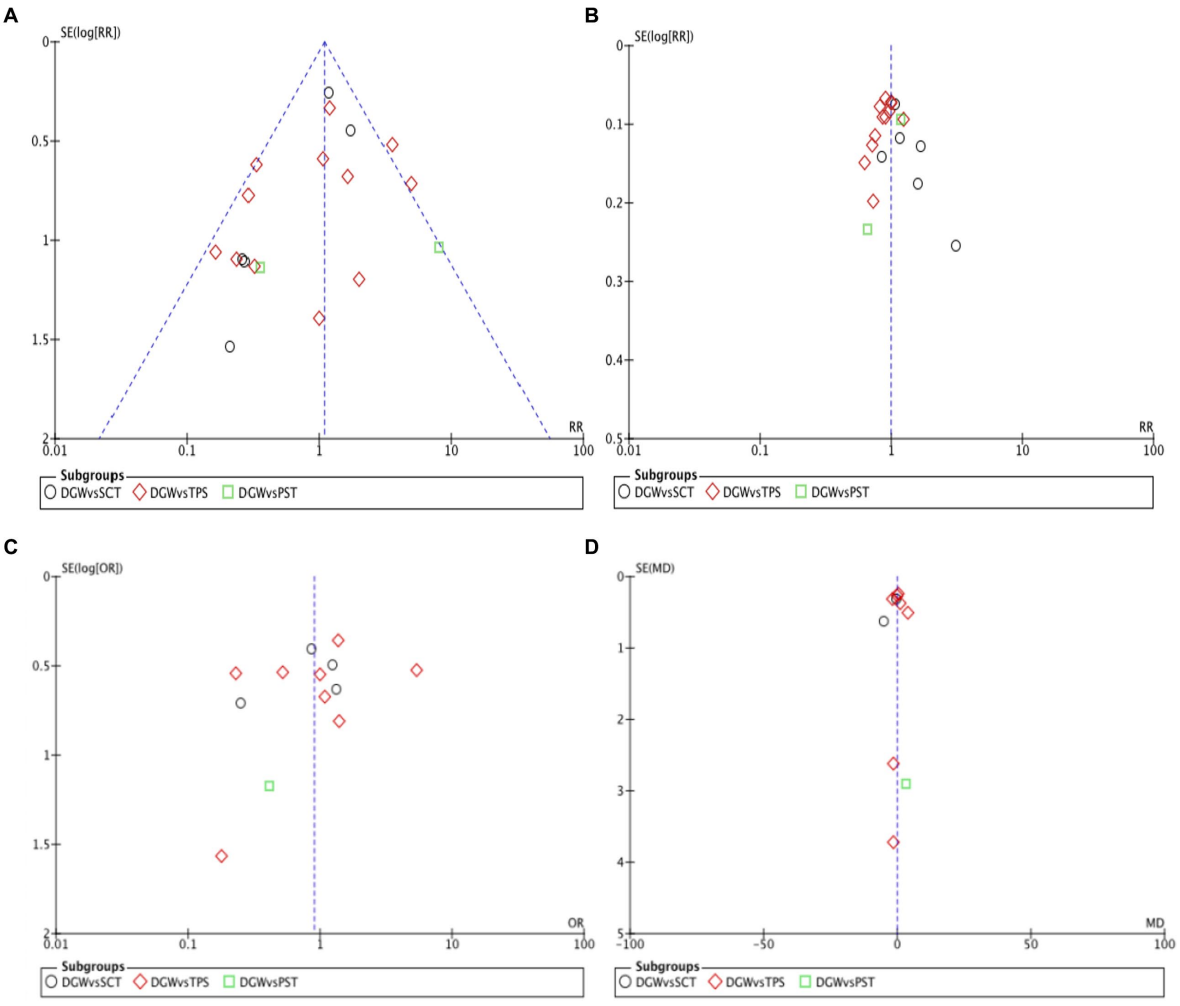
Funnel plot of the cholangiopancreatography (post-ERCP) pancreatitis **(A)**. The success rate of cannulation **(B)**, overall complications **(C)**, as well as the duration of cannulation **(D)** in the double-guidewire technique (DGW) group vs. other endoscopic techniques groups, in patients with difficult cannulation.

Many studies were excluded and analyzed based on the results of PEP, the success rate of cannulation, the duration of cannulation, and overall postoperative complications between DGW and other techniques. The results of this study were consistent with those of studies before exclusion. Additionally, the meta-analysis showed that it does not matter whether the indomethacin suppository was used before the surgery, consistent with previous studies, suggesting the credibility of the meta-analysis. The incidence rate of the success rate of cannulation, duration of cannulation, and overall postoperative complications were analyzed by a funnel plot, which is basically symmetric, suggesting that there was no significant publication bias in the literature (Figure 8).

## 4 Discussion

### 4.1 Interpretation

Over the past 50 years, ERCP has evolved from a tool of diagnosis to a preferred minimally invasive surgical technology for multiple pancreaticobiliary diseases. However, despite significant technological advances, ERCP is still considered a challenging technique for many endoscopy practitioners, largely due to its high rate of cannulation failure, post-ERCP pancreatitis, and other unpredictable complications (26, 27).

There is a close relationship between the occurrence of PEP and ERCP procedure. The PEP can be attributed to many factors, including patients (such as youth, obesity, and female), medical history (sphincter of Oddi dysfunction and PEP history), technical factors (e.g., transpancreatic sphincterotomy, pancreatic duct sphincterotomy, papillectomy, difficult cannulation, and injection of contrast into pancreatic duct), and the experience of operators (28–30). At present, researchers have been committed to reducing the incidence of PEP. Currently, the recommended practice includes guidewire-assisted cannulation, pancreatic stent-assisted technique, preoperative use of indomethacin suppository, and recently proposed active supplementation of lactate ringer after surgery to prevent the PEP in high-risk patients (28, 31, 32).

Reports have shown that the failure rate of selective biliary cannulation was approximately 5–15%, even if operated by experienced endoscopists (33). Cannulation strategy and the duration of the operation determine the success rate of biliary cannulation and the incidence rate of postoperative complications, such as PEP (34, 35). There are still debates on the use of DGW, transpancreatic sphincterotomy, and pancreatic stent-assisted techniques in previous studies (5–24).

This study included 20 studies to explore the differences in ERCP difficult biliary cannulation, the success rate of cannulation, overall postoperative complications, and the duration of cannulation between DGW and other techniques (persistent standard cannulation techniques, transpancreatic sphincterotomy, and pancreatic stent-assisted technique). The results showed that there were no significant differences among the different groups. The meta-analysis published in 2017 by Frances Tse (36) showed that the incidence of PEP in DGW was higher compared with other techniques. Further analysis demonstrated that the incidence of PEP in DGW was only higher than that in transpancreatic sphincterotomy, with no significant difference compared to persistent standard cannulation techniques and pancreatic stent-assisted technique. The study by Frances Tse only included seven studies. In addition, an article published by Antonio Facciorusso (37) in 2022 stated: “Low-quality evidence supported the use of transpancreatic sphincterotomy over persistence with standard cannulation techniques,” and it included a total of 17 articles. Our study also demonstrated that the success rate of cannulation ranked from transpancreatic sphincterotomy followed by the double-guidewire technique to persistent standard cannulation techniques. Theoretically, transpancreatic sphincterotomy performed after the guidewire enters the pancreatic duct, which can not only straighten the common passage of the biliopancreatic duct but also help expose the biliary duct, which is conducive to biliary cannulation.

### 4.2 Limitations

However, there are also limitations to this study. First, the number of subjects included in the literature is small. Second, there is some heterogeneity among different studies. Additionally, the experience of the operators is also related to the postoperative complications of ERCP and the success rate of cannulation. In this study, it is explicitly stated that the operation was performed by experienced endoscopists in seven (7, 10, 14, 15, 22–24) studies, while it has not been mentioned in other studies. Guidelines indicate that the use of the indomethacin suppository and prophylactic pancreatic stent-assisted techniques before ERCP can prevent the development of PEP (32, 38). Among the 20 studies, two (3, 10) studies explicitly suggested the use of indomethacin suppository before surgery to prevent PEP, and three studies (3, 6, 9) mentioned prophylactic pancreatic stent-assisted technique when appropriate or when the duration of cannulation was more than 10 min or 10 attempts.

### 4.3 Conclusion

In conclusion, there was no significance in PEP, overall postoperative complications, and duration of cannulation between the double-guidewire technique and other techniques. The success rates of intubation were, in descending order, transpancreatic sphincterotomy, double guidewire technique, and continuous standard intubation technique.

## Data availability statement

The original contributions presented in the study are included in the article/Supplementary material, further inquiries can be directed to the corresponding authors.

## Author contributions

LiaW: Data curation, Project administration, Resources, Software, Validation, Visualization, Writing – original draft. LimW: Formal analysis, Validation, Writing – original draft. NH: Software, Writing – review & editing. TL: Formal analysis, Methodology, Writing – original draft. XS: Conceptualization, Resources, Writing – review & editing. QZ: Conceptualization, Funding acquisition, Supervision, Writing – review & editing.
